# Supplementary figures and images for: Improving plant miRNA-target prediction with self-supervised k-mer embedding and spectral graph convolutional neural network
Source: PeerJ. 2024 May 21;12:e17396. doi: 10.7717/peerj.17396 (PMC11122044; doi:10.7717/peerj.17396)

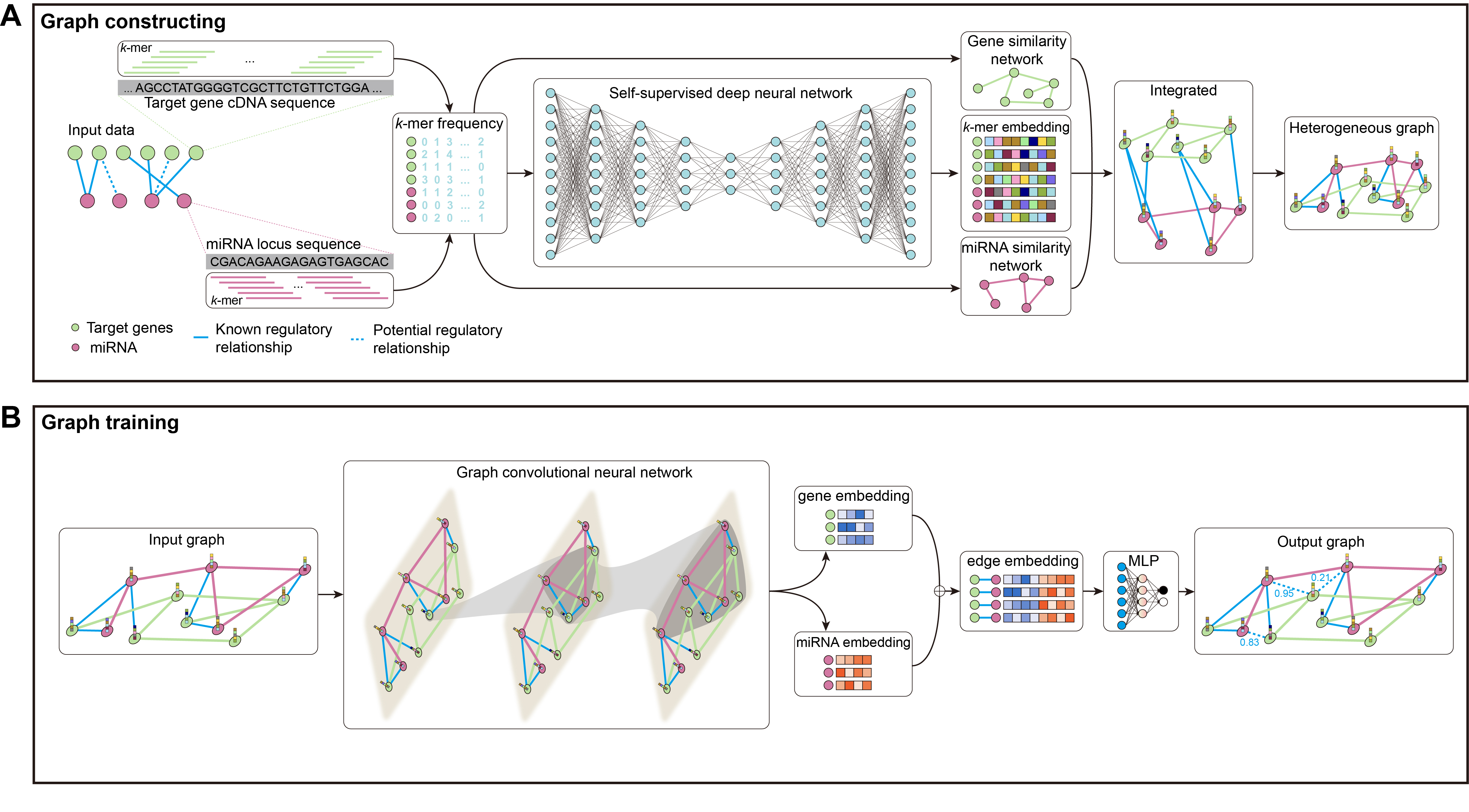

Supplement: Supplemental Information 1 — (A) The graph constructing model. (B) The graph training model. [file peerj-12-17396-s001.jpg]

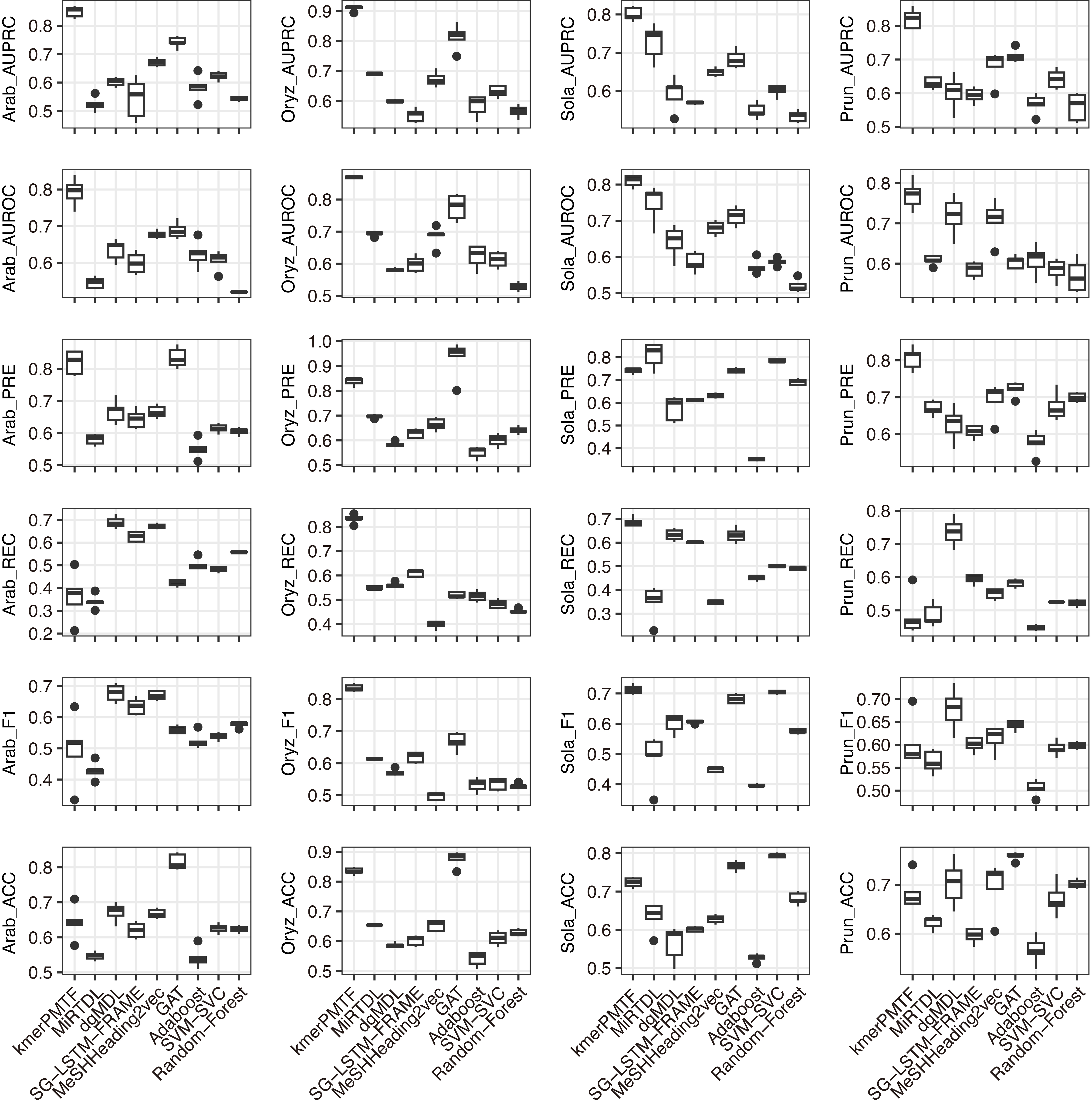

Supplement: Supplemental Information 2 [file peerj-12-17396-s002.jpg]
